# Effect of Rejuvenators on the Workability and Performances of Reclaimed Asphalt Mixtures

**DOI:** 10.3390/ma14216385

**Published:** 2021-10-25

**Authors:** Wei Tang, Xin Yu, Ning Li, Fuqiang Dong, Zhongyuan Wang, Yu Zhang

**Affiliations:** 1 College of Civil and Transportation Engineering, Hohai University, Nanjing 210098, China; tw1125@hhu.edu.cn (W.T.); lining24@hhu.edu.cn (N.L.); hhu_dfq@hhu.edu.cn (F.D.); wang2020@hhu.edu.cn (Z.W.); zzzyyy@hhu.edu.cn (Y.Z.); 2 School of Traffic and Transportation Engineering, Changsha University of Science and Technology, Changsha 410114, China

**Keywords:** RAP, reclaimed asphalt mixture, workability, performance, emulsified rejuvenator

## Abstract

The use of rejuvenators has enhanced the workability of asphalt mixtures containing the reclaimed asphalt pavement (RAP). This conclusion is based on the determination of viscosity of asphalt binders, while not validated from reclaimed asphalt mixtures. In this study, the effect of two rejuvenators (ordinary and emulsified rejuvenator) on the workability of reclaimed asphalt mixtures was evaluated by measuring the mixing torque and determining the air void content of reclaimed mixtures. In addition, their effects on the performances of reclaimed mixture were studied via the three indexes tests, rutting test and freeze-thaw splitting tests. The experimental results show that mixing torque and air void content of reclaimed mixtures with the emulsified rejuvenator is 4% and 6% lower than that with the ordinary rejuvenator, respectively. This indicates that improvement of the workability of reclaimed mixtures can be achieved by using an emulsified rejuvenator, but not by an ordinary rejuvenator. That is also the reason that at least 20% greater high-temperature stability is found for reclaimed mixtures by using the emulsified rejuvenator than using the ordinary rejuvenator. In addition, reclaimed mixtures with the emulsified rejuvenator show similar moisture susceptibility to that with the ordinary rejuvenator. This study provides a feasible method to assess the workability effect of rejuvenators on reclaimed mixtures directly and recommends the use of an emulsified rejuvenator to improve the workability and high-temperature stability of reclaimed mixtures.

## 1. Introduction

The growing attention devoted to environmental conservation and sustainable development, and the utilization of nonrenewable resources, such as oil and minerals, has been limited gradually [1]. That results in the continuous rise in the price of asphalt and aggregates [2]. In China, nearly 790 million tons of reclaimed asphalt pavement (RAP) is produced every year [3,4]. After good regeneration and appropriate mix design, RAP can substitute for the partial asphalt and aggregate in asphalt mixtures [5]. The use of RAP thus has gained more and more popularity due to its environmental and economic benefits [6].

The binder in RAP, however, is highly stiff and viscous after its service life [1]. The asphalt mixtures mixed with RAP are prone to yield fatigue failure and low temperature cracking [7]. Some researchers and engineers recommended using a softer virgin asphalt to recover the properties of aged asphalt to the level that resembles that of virgin asphalt [8,9]. Nevertheless, this method obtains inefficiently little effort when the asphalt binder is heavily aged or more RAP is used [10]. At present, rejuvenators are commonly used in reclaimed mixtures to recover the properties of aged asphalt [11,12]. Therefore, many studies were carried out on the effect of rejuvenators on the properties of aged asphalt and reclaimed mixtures so as to obtain good rejuvenators. It was reported by Zaumanis et al. that the waste vegetable oil-based rejuvenator can largely reduce the performance grade and fatigue parameters of aged asphalt compared to six other types of rejuvenators [13,14]. Waste engine oil can also be used as a rejuvenator and it is found to enhance the mechanical properties and moisture resistance of reclaimed mixtures [15,16]. Furthermore, the use of waste polymer modified asphalt in combination with rejuvenators can significantly improve the fatigue, rutting and moisture resistance of reclaimed mixtures [17]. Rejuvenators can diffuse into aged asphalt resulting in the improvement of the physical properties of aged asphalt, such as being more permeable and softer. It is believed that the rejuvenating effect of rejuvenators is largely dependent on the diffusibility of the rejuvenator into aged asphalt [18]. It is found that the diffusibility of rejuvenators increases with increasing temperature and time [19]. Additionally, rejuvenators with low viscosity diffuse into aged asphalt with more ease.

Based on the effect of rejuvenators on aged asphalt, it seems that the use of rejuvenators can improve the workability of reclaimed asphalt mixtures [20]. It was reported by Ahmad et al. that the vegetable oil-based rejuvenator improved the workability of reclaimed asphalt mixtures due to the reduction in viscosity of aged asphalt [21]. Bio-based rejuvenators, which are refined from cooking oil waste, can also enhance the workability of reclaimed asphalt mixtures [8,22]. It should be noted that the conclusion about the improvement of rejuvenators on the workability of reclaimed asphalt mixtures is based on the determination of the viscosity of the asphalt binder. From the aspect of practical application, it is better to discuss the effect of the rejuvenator on the properties of reclaimed asphalt mixtures directly. In engineering practice, the rejuvenator is required to be heated at relatively high temperature for usage, which will result in the volatilization of the light component and aging of the rejuvenator [19]. If the rejuvenator is emulsified, it can be used without heating and maybe improve the workability of reclaimed mixtures. Therefore, this study was carried out to discuss the effect of rejuvenators on the workability and performances of reclaimed asphalt mixtures. An emulsified rejuvenator was investigated and compared with an ordinary rejuvenator. The workability was evaluated via measuring the torque generated when mixing reclaimed mixtures and determining the air void content of the compacted mix. The performances of reclaimed mixtures include high-temperature stability and moisture susceptibility.

## 2. Materials Preparation

### 2.1. Raw Materials

**Figure 1:**
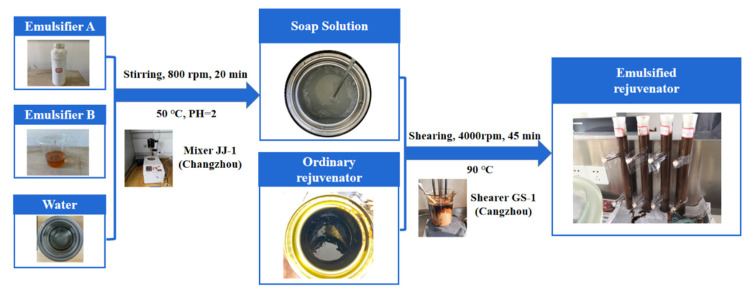
Flowchart of preparing ER.

An ordinary rejuvenator meeting ASTM D4552 specification was selected as the control rejuvenator (CR). Table 1 shows the properties of the control rejuvenator. Using this control rejuvenator as a base component, an emulsified rejuvenator (ER) was self-developed and used in this study. Table 2 shows the properties of the emulsifiers used. The detailed preparation procedure of ER is described in Figure 1. The water content by mass of ER was 40%.

**Figure 2:**
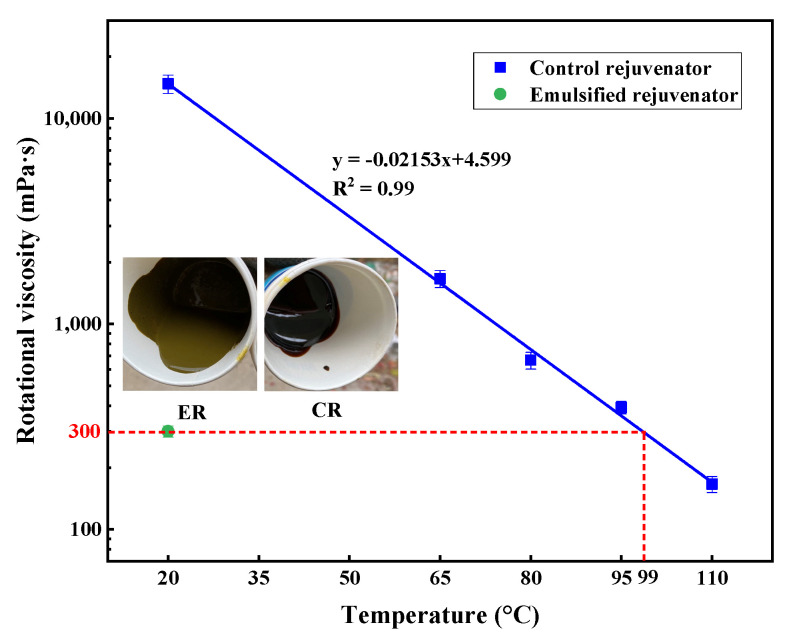
Rotational viscosity of ER and CR.

Figure 2 presents the images of the two rejuvenators and their rotational viscosity values at different temperatures. It can be visually found from the images that the ER shows better flowability than the CR at room temperature. The rotational viscosity of ER at 20 °C was 300 mPa·s, dramatically lower than that of CR at the same temperature. Therefore, it is believed that ER can be used without heating in engineering practice. Styrene-butadiene-styrene (SBS) modified asphalt binder, PG 76-22, was used as the virgin asphalt and the fundamental material to produce laboratory-aged asphalt. The rotational viscosity of this modified asphalt at 135 °C was 2690 mPa·s and the G*/sinδ at 76 °C was 2.26 kPa. These tests were performed according to ASTM D4402 and AASHTO T315.

The RAP material was obtained from the upper layer of a freeway which has already been serviced for 11 years. After preprocessing, the RAP material was divided into three groups: 10~16 mm, 5~10 mm, and 0~5 mm. According to ASTM D2172, the asphalt content of RAP with corresponding size is determined as 2.3%, 1.6% and 7.0%, respectively. In addition, the gradations of extracted aggregates are determined and presented in Table 3. The virgin aggregates were basalt and mineral filler was limestone.

### 2.2. Preparation of Recycled Asphalt Binders

Recycled asphalt binders were prepared to evaluate the regeneration effect of the rejuvenators on aged asphalt. Prior to preparing recycled asphalt, laboratory-aged asphalt was first obtained via aging the SBS-modified asphalt in a rolling thin film oven (RTFO) at 163 °C for 5 h [23]. This method allows for sufficient aged asphalt to be collected within a short period of time. After that, preparation of recycled asphalt binder was accomplished by blending aged asphalt, virgin asphalt and rejuvenator together at 175 °C for 2 h. The weight ratios of aged asphalt to total recycled asphalt binder were 30%, 50% and 100%. It is noted herein that CR and ER was added in accordance with different contents. The contents of CR and ER were set at 4% and 6.67% by weight of aged asphalt, respectively, which was done to ensure equal weight of pure rejuvenator in recycled asphalt.

### 2.3. Mix Design of Reclaimed Asphalt Mixtures

**Figure 3:**
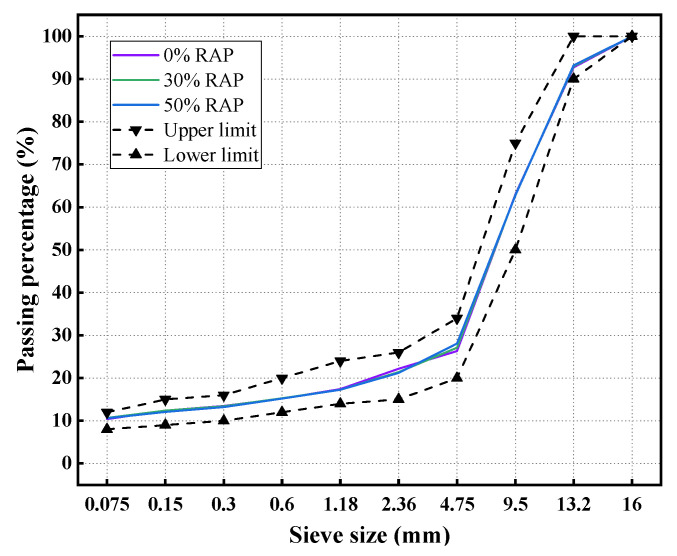
Combined gradations of reclaimed asphalt mixtures.

With standard SMA-13 gradation, the reclaimed asphalt mixtures were designed for workability and performance tests. The RAP content in the reclaimed mixtures was 0% (virgin asphalt mixtures), 30% and 50%, respectively. As depicted in Figure 3, the combined gradations of those asphalt mixtures were designed as close as possible to eliminate the interference of gradation. Based on Marshall mix design, the asphalt contents were determined as 6.0% for these reclaimed mixtures (see Table 4).

## 3. Evaluation Methods

### 3.1. Workability Evaluation Methods

The rotational viscosity of asphalt binder is commonly used to determine the mixing and compaction temperature of asphalt mixtures [24]. Similarly, the workability of asphalt mixtures is also reflected in the mixing and compaction process [25]. In this study, the workability in the two process was assessed by measuring mixing torque and determining air void content of compacted mix, respectively.

#### 3.1.1. Measurements of Mixing Torque

**Figure 4:**
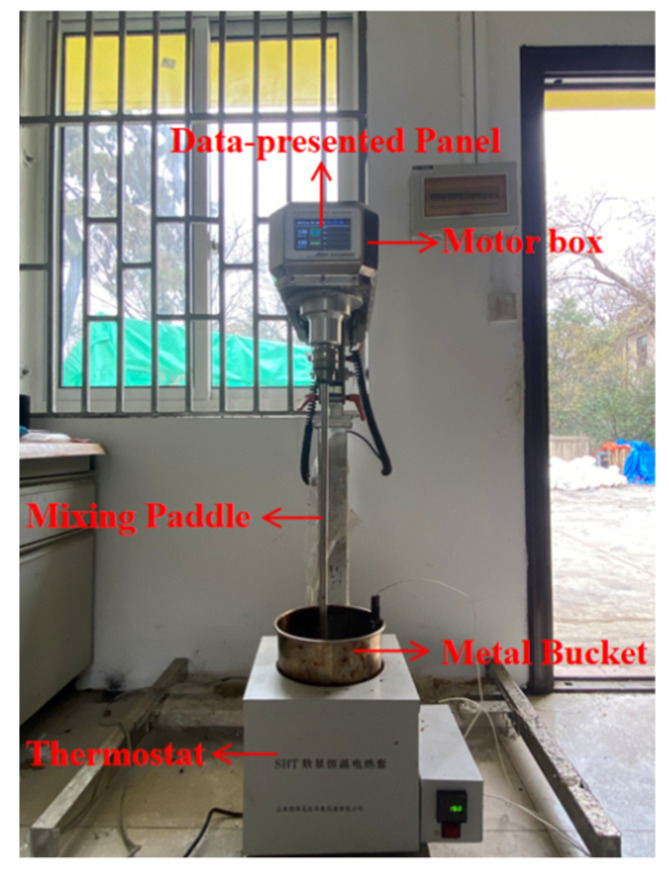
Mixing torque tester.

It is confirmed in many previous studies that the torque generated when stirring asphalt mixtures is highly related to the mixing workability of mixtures [26,27,28]. The lower mixing torque means the better workability of asphalt mixtures. In this study, the mixing torque was used to characterize the workability of reclaimed asphalt mixtures. A device that can measure the torque was self-fabricated and used. Figure 4 shows a photograph of this device, which mainly consists of (1) motor box (ANGNI, AN1000, Shanghai, China), (2) mixing paddle, (3) metal bucket, and (4) thermostat. The motor allows the device to stir at different speeds. Importantly, the device includes a data-acquisition system that enables it to record the torque readings automatically.

The reclaimed mixtures with RAP content of 30% was selected for this test and virgin mixtures were used as the control group. According to trial and error, each batch of asphalt mixtures for testing weighed 800 g approximately, which ensured the stable data during mixing process. The mixing temperatures were set at 130 °C, 150 °C and 170 °C. Prior to testing, the mixtures were heated 5 °C higher than mixing temperature in an oven. Upon completing the heating step, the mixtures and rejuvenator were placed into the metal bucket. The mixing paddle started to rotate at a constant speed of 100 r/min. Meanwhile, data-acquisition system was lunched to record the torque values. The test lasted approximately 3 min in general.

#### 3.1.2. Determination of Air Void Content

There is a method of evaluating the workability of asphalt mixtures in compaction process, which is determined with the use of air void content of specimens produced with Marshall compactor [25,29]. The better workability of asphalt mixtures can be obtained if the air void content is lower. In this study, Marshall specimens were firstly prepared following mix design in Table 4. Then, the air void contents of these specimens were tested to assess the workability. The detailed procedures are as follows.

1. The RAP, virgin aggregates and virgin asphalt were heated at 140 °C, 180 °C and 170 °C for 2 h, respectively. The CR was heated to 110 °C, while the CR was used at room temperature without heating;
2. The RAP was premixed with rejuvenator in a laboratory mixer (model F-20, Changji, Shanghai, China). The virgin aggregates, virgin asphalt and powder were successively added into the mixer for mixing. Each mixing duration was 60 s;
3. After mixing, the loose reclaimed mixtures were subjected to heat preservation in an oven. The preserved temperature was 160 °C and preserved time was 1 h;
4. Marshall specimens were prepared with 75 blows per side following the Chinese specification JTG-E20 T0702. Lastly, surface-dry method was used to measure the air void content.

### 3.2. Performances Evaluation Methods

#### 3.2.1. Three Indexes Tests

Three indexes of asphalt binder refers to the softening point, the penetration and the ductility. To evaluate the regeneration effect of rejuvenator on the aged asphalt, three indexes tests were performed on the recycled asphalt binders. These test methods referred to the ASTM D36, D5, and D113, respectively.

#### 3.2.2. Rutting Test

The rutting test was performed in accordance with JTG-E20 T0719 to evaluate the high-temperature stability of reclaimed mixtures [24]. The test temperature was 60 °C and the wheel pressure was 0.7 MPa. After testing, the dynamic stability (DS) was determined to evaluate the high-temperature stability.

#### 3.2.3. Freeze-Thaw Splitting Tests

It is essential to evaluate the moisture susceptibility of reclaimed asphalt mixtures when using emulsified rejuvenator, since moisture is induced into the rejuvenator. In this study, the freeze-thaw splitting test was used to assess the moisture susceptibility of asphalt mixtures. This test was conducted according to JTG-E20 T0729. Marshall specimens (50 blows for each side) were prepared and then equally divided into dry group and freeze-thaw group. The splitting test was performed for each group to determine tensile strength ratio (TSR), viz. the evaluation index of moisture susceptibility.

## 4. Results and Discussions

### 4.1. Effect of Rejuvenators on the Workability of Reclaimed Mixtures

#### 4.1.1. Mixing Torque

**Figure 5:**
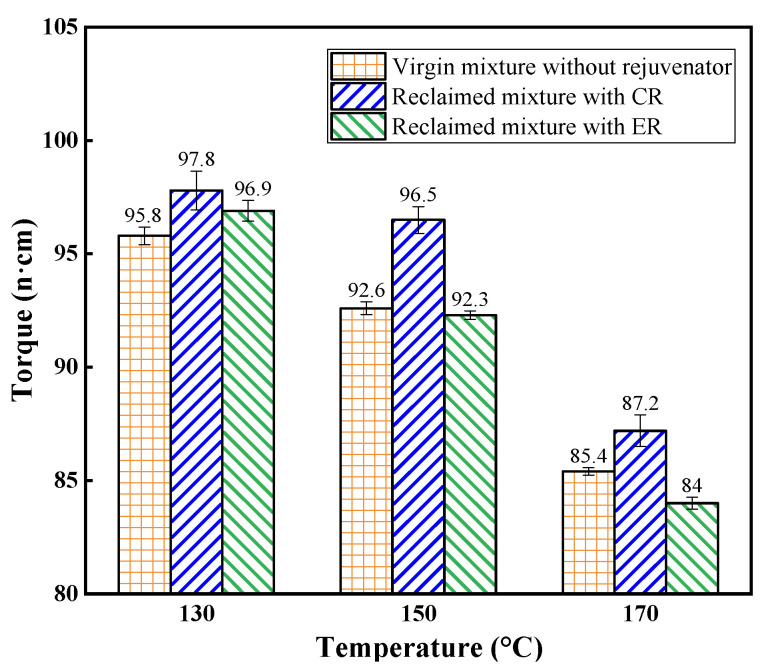
Torque values of asphalt mixtures at different mixing temperatures.

The torque values are presented in Figure 5. As expected, the torque values decreased with the increase of temperature due to the reduction in the viscosity of the asphalt binder. The reclaimed mixtures with CR exhibited higher torque values than the virgin mixtures at the same testing temperatures. The higher torque values for reclaimed mixtures can be attributed to the aged asphalt in RAP, which was more stiff and viscous than the virgin asphalt. This also gives a reasonable explanation as to why production temperature for reclaimed asphalt mixture is required to be 5~10 °C higher than that for virgin mixtures in Chinese specification JTG T55-2019 [30].

By comparing the torque values obtained from the reclaimed asphalt mixtures, it can be seen that reclaimed mixtures with ER showed lower torque values than that with CR, indicating that ER helped to improve the workability of reclaimed mixtures. This can be due to that compared to oil mediums, the oil-water emulsified film showed better lubricating effect during the mixing process [31]. The resistance between aggregates particles, thus, was reduced. Furthermore, the greater difference in torque values for the reclaimed mixtures at higher temperature (150 °C and 170 °C) can be obtained. The torque values for reclaimed mixtures with ER were even lower compared to virgin mixtures.

#### 4.1.2. Air Void Content

**Figure 6:**
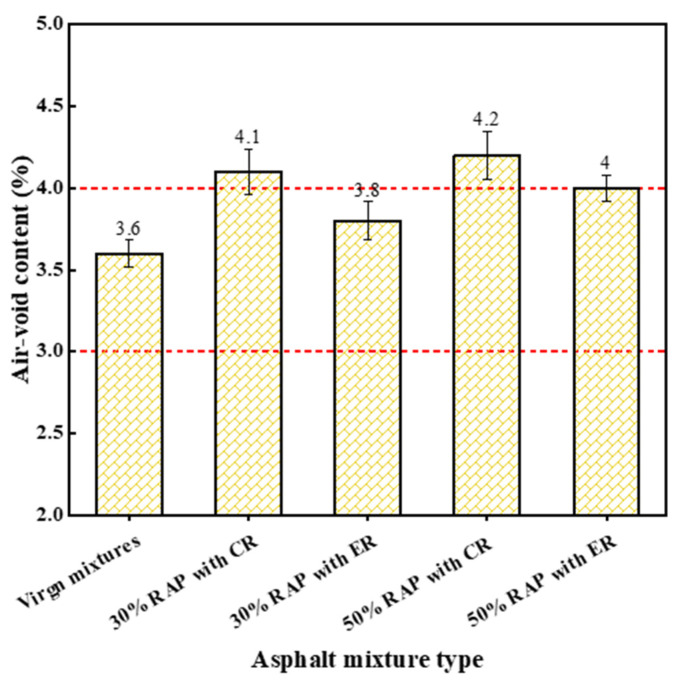
Air void content of each studied mixture.

Figure 6 presents the air void content of the Marshall specimen. The air void content of virgin mixtures was within the limit of 3~4%, while that of reclaimed mixtures with CR exceeded the upper limit. This implies that the addition of RAP in asphalt mixtures exhibited an adverse effect on the workability in the compaction process. By comparing the air void contents between the reclaimed mixtures produced with CR and ER, it can be found that an approximately 6% degree of workability was improved by using ER. These findings are consistent with the results showed by the mixing torque test. Unlike mixing workability, the workability in the compaction process of reclaimed mixtures produced with ER did not exceed the level of that of the virgin mixtures. That result can be seen from the comparison of air void contents of the two mixtures.

### 4.2. Effect of Rejuvenators on the Performances of Reclaimed Mixtures

#### 4.2.1. Three Indexes of Recycled Asphalt Binders

**Figure 7:**
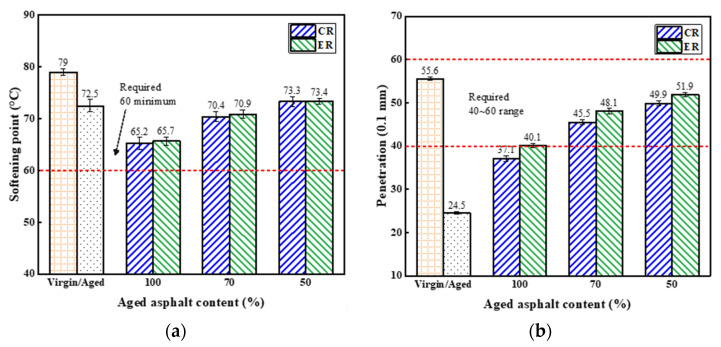

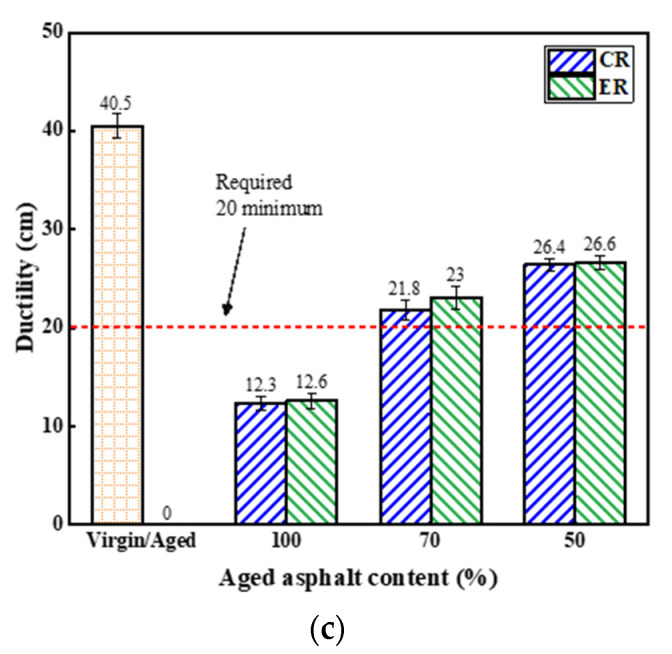
Three indexes of different asphalt binders: (**a**) softening point; (**b**) penetration; (**c**) ductility.

Test results of three indexes of different asphalt binders are shown in Figure 7. It was found that after long-term aging, the softening point, penetration and ductility of virgin asphalt all decreased. With the incorporation of rejuvenators into the aged asphalt, the softening point decreased, and the penetration and ductility increased. This indicates the performances of the aged asphalt had been partially recovered. However, three indexes of recycled asphalt were difficult to meet the requirements in JTG-F40 specification [32] simultaneously when only rejuvenator was added. In the case of the addition of rejuvenator and virgin asphalt, aged asphalt content can reach to 70% in view of the fact that these indexes of recycled asphalt all satisfied the requirements. In addition, the values of three indexes of recycled asphalt with ER were equivalent to that of recycled asphalt with CR. This may be attributed to complete dissipation of moisture during the preparation process of recycled asphalt or little residual water that basically had no effect on the properties of recycled asphalt [33].

#### 4.2.2. High-Temperature Stability

**Figure 8:**
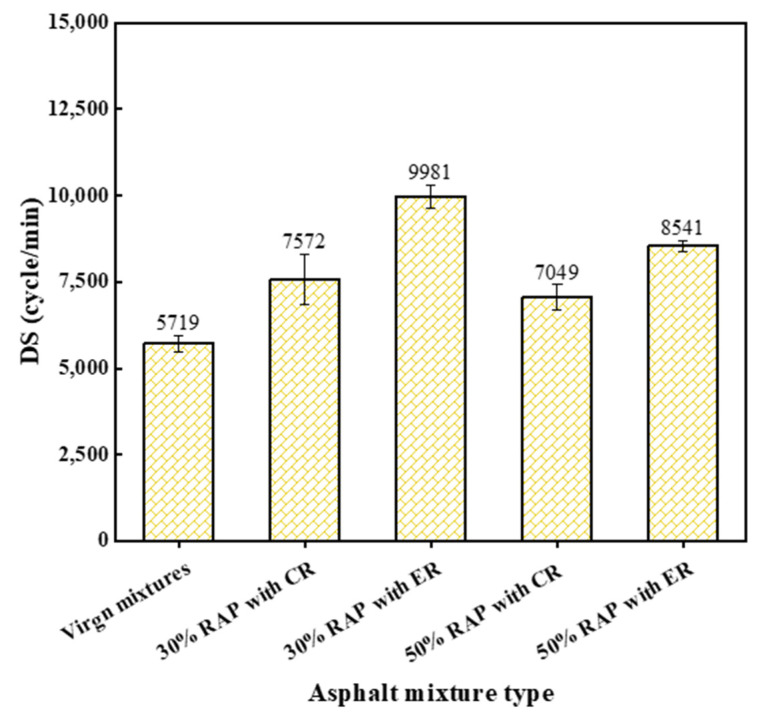
The dynamic stability (DS) results of each studied mixture.

The dynamic stability (DS) results of each studied mixture are presented in Figure 8. The higher DS means the better high-temperature stability. As can be found in this figure, the DS values of all reclaimed mixtures were higher than that of the virgin mixture. However, the DS values of reclaimed mixtures did not vary monotonously with the increasing RAP content. The sample with 30% RAP content had the highest DS. Among these reclaimed mixtures, the mix with CR showed lower DS than the mix with ER, indicating that using ER in reclaimed mixtures results in better high-temperature stability. It was found above that the use of ER improved the workability of reclaimed mixtures, which results in lower air void content in reclaimed mixtures (see Figure 6). This may be the reason why better high-temperature stability of reclaimed mixture with ER was obtained.

#### 4.2.3. Moisture Susceptibility

**Figure 9:**
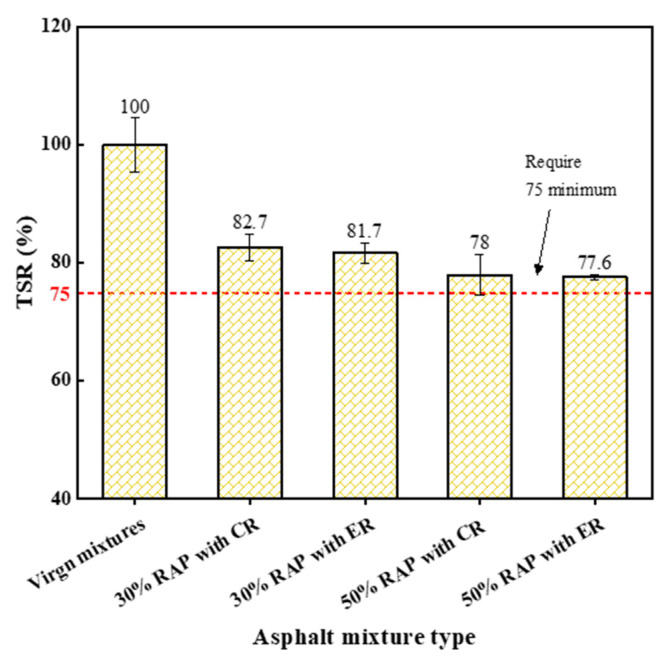
The tensile strength ratio (TSR) results of each studied mixture.

Figure 9 shows the tensile strength ratio (TSR) of the mixtures. Obviously, the TSR of reclaimed mixtures was lower than that of virgin mixtures, indicating that the use of RAP in asphalt mixtures is adverse to the moisture sensibility. There are two probable factors contributing to the worse moisture susceptibility of reclaimed mixtures. The first is that low surface free energy of aged asphalt weakens the bond between aggregates and aged asphalt [34]. The aged asphalt strips from the RAP particles with more ease under the freeze-thaw condition, which results in the water damage. The second can be that the incomplete blending between virgin asphalt and aged asphalt yields a weak interface, and water damage tends to occur at this interface easily [35]. The reclaimed mixtures with ER exhibited the nearly equivalent TSR to that with CR, although water was induced in ER. Overall, all studied mixtures had good moisture susceptibility, because these specimens showed TSR values beyond the requirements in Chinese JTG-F40 specification.

## 5. Conclusions

The effects of an ordinary rejuvenator and self-developed emulsified rejuvenator on the workability of reclaimed asphalt mixtures were compared by measuring the mixing torque and determining the air void content. Additionally, their effects on the high-temperature stability and moisture susceptibility of asphalt mixtures were evaluated. Based on the test results, some major conclusions can be drawn as follows.

i. The emulsified rejuvenator can be used without heating in engineering practice owing to its low viscosity, while the ordinary rejuvenator has to be heated.
ii. The addition of RAP into asphalt mixtures has an adverse effect on the workability. Compared to using an ordinary rejuvenator, reclaimed mixtures prepared using the emulsified rejuvenator exhibit lower torques and air void contents. This can be attributed to that using oil-water emulsified medium shows better lubricating effect during the mixing and compaction process.
iii. Reclaimed mixtures with the emulsified rejuvenator show similar moisture susceptibility to that with the ordinary rejuvenator. At least 20% higher high-temperature stability is obtained for reclaimed mixtures by using the emulsified rejuvenator than an ordinary rejuvenator. This can be attributed to better workability when using the emulsified rejuvenator, which results in lower air voids content in the reclaimed mixtures.
iv. The emulsified rejuvenator improves the workability and high-temperature stability of reclaimed mixtures, while not affecting the moisture susceptibility of mixtures. Therefore, rejuvenators are recommended to be emulsified for use in practice.

## Figures and Tables

**Table 1:** Properties of CR used in this study.

Properties	Test Results	Requirements (RA-25)	Specification Method
Viscosity, mm^2^/s (60 °C)	2080	901~4500	ASTM D2171
Flash point, °C	240	>219	ASTM D92
Saturates, % (wt)	22.3	<30	ASTM D2007
Wt change, % (After TFOT, 163 °C)	0.5	<3	ASTM D1754
Specific gravity	0.99	Report	ASTM D70

**Table 2:** Properties of emulsifier used in this study.

Properties	Emulsifier A	Emulsifier B
Appearance	viscous liquid	yellow liquid
Electric charge	+	+
Blend stability	quick-set	quick-set
PH of aqueous solution	10	11.3
Amine value (mg/g)	-	360–410

**Table 3:** Gradations of extracted aggregates.

RAP Size (mm)	Passing Percentage of Different Sieve Size/%									
	16	13.2	9.5	4.75	2.36	1.18	0.6	0.3	0.15	0.075
10~16	100	89.3	32.8	10.5	9.1	7.6	6.4	4.8	3.9	3.4
5~10	100	100	91.2	18.6	11.0	8.8	7.5	6.2	5.4	4.8
0~5	100	100	100	90.9	62.4	41.0	29.6	19.6	15.8	13.7

**Table 4:** Mix design results.

RAP (%)	Virgin Mineral Materials (%)				AC (%)	Rejuvenator ^1^ (%)
	10~15 mm	5~10 mm	0~3 mm	Filler		
0	39.8	31.8	12.1	10.3	6.0	0
30	34.0	18.0	4.3	8.8	6.0	4/6.67
50	30.1	8.3	0	7.3	6.0	4/6.67

^1^ Rejuvenator: 4% and 6.67% was the content of control rejuvenator and emulsified rejuvenator by weight of aged asphalt, respectively.

## Data Availability

No new data were created or analyzed in this study. Data sharing is not applicable to this article.
